# Potential Costs of Acclimatization to a Warmer Climate: Growth of a Reef Coral with Heat Tolerant vs. Sensitive Symbiont Types

**DOI:** 10.1371/journal.pone.0010437

**Published:** 2010-05-03

**Authors:** Alison Jones, Ray Berkelmans

**Affiliations:** 1 Centre for Environmental Management, Central Queensland University, Rockhampton, Queensland, Australia; 2 Australian Institute of Marine Science, Townsville, Queensland, Australia; American Museum of Natural History, United States of America

## Abstract

One of the principle ways in which reef building corals are likely to cope with a warmer climate is by changing to more thermally tolerant endosymbiotic algae (zooxanthellae) genotypes. It is highly likely that hosting a more heat-tolerant algal genotype will be accompanied by tradeoffs in the physiology of the coral. To better understand one of these tradeoffs, growth was investigated in the Indo-Pacific reef-building coral *Acropora millepora* in both the laboratory and the field. In the Keppel Islands in the southern Great Barrier Reef this species naturally harbors nrDNA ITS1 thermally sensitive type C2 or thermally tolerant type D zooxanthellae of the genus *Symbiodinium* and can change dominant type following bleaching. We show that under controlled conditions, corals with type D symbionts grow 29% slower than those with type C2 symbionts. In the field, type D colonies grew 38% slower than C2 colonies. These results demonstrate the magnitude of trade-offs likely to be experienced by this species as they acclimatize to warmer conditions by changing to more thermally tolerant type D zooxanthellae. Irrespective of symbiont genotype, corals were affected to an even greater degree by the stress of a bleaching event which reduced growth by more than 50% for up to 18 months compared to pre-bleaching rates. The processes of symbiont change and acute thermal stress are likely to act in concert on coral growth as reefs acclimatize to more stressful warmer conditions, further compromising their regeneration capacity following climate change.

## Introduction

Coral reefs are generally thought to be highly vulnerable to climate change as they live in a narrow range of thermal tolerance. Recent research however, indicates that scleractinian (reef-building) corals may have considerable scope for acclimatization to warmer conditions [1], [2], [3]. The key to acclimatization may be their capacity to ‘shuffle’ the levels of symbiotic zooxanthellae genotypes (taxonomic units below the clade level) that are now known to occur within the tissues of most corals [4], [5]. Symbiont shuffling occurs when the relative dominance of symbiont types changes. Under temperature stress, thermally sensitive symbiont types are displaced in favor of thermally tolerant types [6]. For instance, changing to thermally tolerant *Symbiodinium* type D in one study was found to increase thermal tolerance between 1.0–1.5°C in a common Indo-Pacific coral species, *A. millepora* [7]. Symbiont change on reefs must essentially involve a community shift in the symbionts of multiple coral species to realize an increase the thermal tolerance of the entire reef [3]. Field studies have yet to demonstrate how widespread the phenomenon of shuffling is, whether all corals have the ability to shuffle symbiont types, or what ecological benefits may result from ‘new’ host-symbiont combinations.

The symbiont type harbored by reef corals can influence the nutritional status and overall fitness of the holobiont. Corals rely heavily on their symbionts for their energy requirements through the translocation of photosynthetically fixed carbon (estimated to be as high as ∼95% of the total energy requirement) [8], [9]. In zooxanthellate corals, some of this energy is used to drive carbonate accretion [10]. This deposition of carbonate (calcification) is the process by which reef-builders form their hard skeletons. Processes that affect photosynthesis have the potential to have a simultaneous effect on host calcification [11]. The link between symbiont genotype, photosynthetic function and carbon fixation has already been established. For instance, Cantin *et al.* [12] found a positive correlation between the tissue incorporation of radio-labeled carbon (which represents photosynthetically-derived carbon-based energy) and the relative maximum rate of electron transport through photosystem II (rETR_max_, a secondary measure of photosynthetic function) in *A. millepora* juveniles with C1 and D symbionts. Type D juveniles had lower rETR_max_ and fixed less ^14^C than those with type C1. Because photosynthesis is directly related to the amount of energy available to the host for calcification, this is likely to reflect in lower calcification and skeletal growth rate. This is supported by studies of growth in juvenile *A. millepora* in which increased skeletal growth has been demonstrated in type C1 compared to type D symbionts [13], [14]. Clearly, predominant symbiont genotype can influence host physiology. Symbiont community change by shuffling therefore has the potential to influence the growth dynamics of entire reef communities. While these studies show that symbiont type can affect the growth of juvenile corals, differences in the growth rates of adult corals with thermo-tolerant and –sensitive zooxanthellae types have so far not been studied.

The growth rate of reef-building corals has a substantial influence on the resilience and regeneration capacity of tropical reefs. The hard skeletons of scleractinian corals form the framework of reefs, providing food and habitat for other marine organisms [15]. Following disturbance, such as bleaching, the growth rate of the key structural corals, such as species belonging to the genera *Acropora* and *Pocillopora*, is the most important factor in reef recovery [16], [17]. Frequent disturbance without strong coral re-growth results in phase shifts to macro-algal and soft coral-dominated communities [18]. The growth rate of hard coral species is an important factor in preventing these phase shifts [19]. Factors that affect the process of calcium carbonate accretion in structural corals, such as *Pocillopora* and *Acropora*, have downstream influence on the habitat and food supply of other marine organisms [20]. The warmer and potentially more acidic marine conditions that are predicted to occur with climate change pose an as yet unquantifiable threat to the carbonate structure of coral reefs [21]. Retardation of coral re-growth by symbiont change could exacerbate these processes, accelerating the demise of coral reefs as we know them.

This study investigates one of the most important elements of reef resilience to climate change, namely skeletal growth. *A. millepora* was chosen for this study as this is an abundant and dominant reef-builder on the leeward shores of islands in the Keppel region of the Great Barrier Reef [6], [22]. Colonies on these reefs naturally host thermally-sensitive type C2 *Symbiodinium*, thermally-tolerant type D or a combination of both types. The results indicate that the growth rate of *A. millepora* is significantly affected in hosts containing the thermally tolerant symbiont type compared to those with the thermally sensitive type but that the stress of the bleaching confounds any costs or benefits of symbiont type.

## Results

### Laboratory study

**Figure 1:**
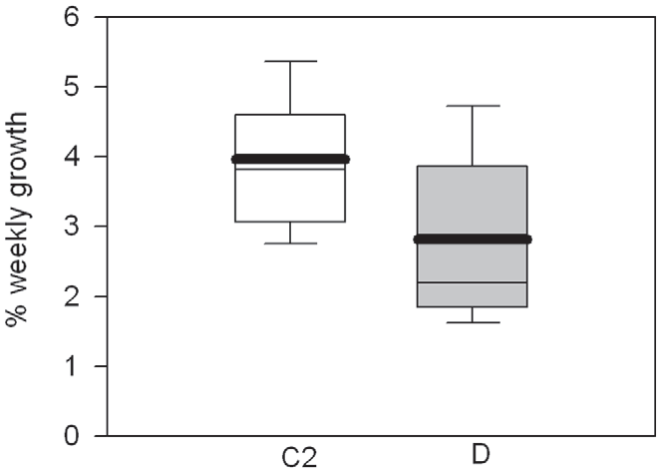
Growth rates of *Acropora millepora* explants in the laboratory. *Acropora millepora* explants with type D symbionts had significantly lower growth than explants with type C2 symbionts. Boxes represent the buoyant weight gained over a week expressed as a percentage of the initial buoyant weight of the explant. White boxes represent colonies with type C2 symbionts and grey boxes represent colonies with type D symbionts. Box boundaries represent the 75th and 25th percentiles. Thick lines within the boxplots represent the mean and thin lines represent the median. Whisker bars above and below the boxes represent the 95th and 5th percentiles.

The skeletal growth rate of *A. millepora* explants in the laboratory varied significantly with predominant symbiont genotype. The buoyant weight gain of explants with type D symbionts for the four weeks of the study was 29% less than that gained by C2 explants (p<0.05, Figure 1, Table 1). There was no significant effect of temperature on explant growth and no significant interaction between symbiont type and temperature treatment.

**Table 1:** ANOVA of the growth rate of *Acropora millepora* with C2 and D symbionts.

	SS	df	Mean Square	F	Sig.
Corrected Model	47.69^a^	3	15.90	8.3	0.000
Intercept	1449.72	1	1449.72	760.1	0.000
Type	41.17	1	41.17	21.6	0.000
Temperature	4.30	1	4.30	2.3	0.136
Type * Temperature	3.01	1	3.01	1.6	0.211
Error	236.51	124	1.91		
Total	1819.63	128			
Corrected Total	284.20	127			

a. R^2^ = 0.168 (Adjusted R^2^ = 0.148).

**Figure 2:**
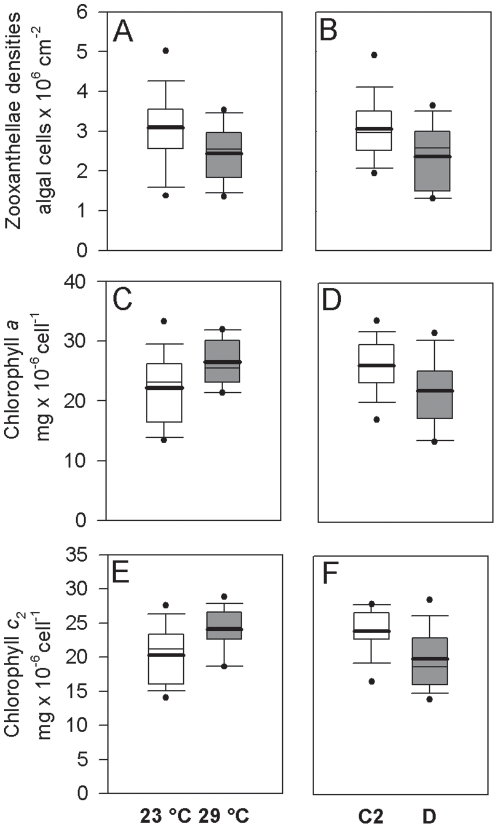
Algal densities and chlorophyll pigments for *Acropora millepora* in the laboratory. Boxplots showing the significant effects of (a, c, e) temperature on zooxanthellae densities and chlorophyll *a* and *c_2_*, and (b, d, f) symbiont genotype on zooxanthellae densities and chlorophyll *a* and *c_2_*, in *Acropora millepora* explants during a laboratory growth experiment. White boxes represent colonies with type C2 symbionts or those at 23°C and grey boxes represent colonies with type D symbionts or those at 29°C. Box boundaries represent the 75th and 25th percentiles. Thick lines within the boxplots represent the mean and thin lines represent the median. Whisker bars above and below the boxes represent the 95th and 5th percentiles. Missing and out of range values are not shown. Dots represent data that fall outside the confidence limits.

Chlorophyll and zooxanthellae density measurements varied significantly with the predominant symbiont genotype and with treatment temperature but there were no significant interactions (Figure 2 a–f, Table 2). Zooxanthellae densities for type D explants (averaged across temperature treatments) were 22% lower than densities for C2 explants (p<0.05). Zooxanthellae densities at 29°C were 21% lower than densities at 23°C (p<0.05). The algal cell chlorophyll *a* content of type D explants was 16% lower than for type C2 explants (p<0.05) while chlorophyll *c_2_* for type D explants was 17% lower (p<0.05). At 29°C, the mean algal chlorophyll *a* was 20% higher than concentrations at 23°C (p<0.05) while chlorophyll *c_2_* was 19% higher (p<0.05). At the end of the laboratory study, zooxanthellae densities and algal cell chlorophyll *a* and *c_2_* compared well with values measured in nearby colonies sampled in the field at Miall Island (data not shown). Fv/Fm measurements of C2 and D colonies were stable between 0.6 and 0.8 throughout the experiment indicating that there was no measureable photo-damage and/or photo-inhibition [23], [24], [25], [26], [27], [28], [29].

**Table 2:** ANOVA of symbiont densities and algal chlorophyll *a* and *c_2_* for *Acropora millepora* in the laboratory.

	Dependent Variable	SS	df	Mean Square	F	Sig.
Corrected Model	Zooxanthellae	7.218^a^	3	2.41	3.6	0.025
	Chlorophyll *a*	348.15^b^	3	116.05	5.2	0.005
	Chlorophyll *c_2_*	262.35^c^	3	87.45	8.0	0.001
Intercept	Zooxanthellae	233.38	1	233.38	349.9	0.000
	Chlorophyll *a*	18275.94	1	18275.94	822.0	0.000
	Chlorophyll *c_2_*	15162.09	1	15162.09	1387.3	0.000
Type	Zooxanthellae	3.77	1	3.77	5.7	0.025
	Chlorophyll *a*	142.50	1	142.50	6.4	0.017
	Chlorophyll *c_2_*	130.76	1	130.76	12.0	0.002
Temp	Zooxanthellae	3.21	1	3.21	4.8	0.037
	Chlorophyll *a*	174.25	1	174.25	7.8	0.009
	Chlorophyll *c_2_*	125.34	1	125.34	11.5	0.002
Type * Temp	Zooxanthellae	0.07	1	0.07	0.1	0.753
	Chlorophyll *a*	51.99	1	51.99	2.3	0.137
	Chlorophyll *c_2_*	15.07	1	15.07	1.4	0.250
Error	Zooxanthellae	18.67	28	0.67		
	Chlorophyll *a*	622.57	28	22.23		
	Chlorophyll *c_2_*	306.02	28	10.93		
Total	Zooxanthellae	270.57	32			
	Chlorophyll *a*	19948.86	32			
	Chlorophyll *c_2_*	16330.80	32			
Corrected Total	Zooxanthellae	25.89	31			
	Chlorophyll *a*	970.72	31			
	Chlorophyll *c_2_*	568.38	31			

a. R^2^ = 0.279 (Adjusted R^2^ = 0.202).

b. R^2^ = 0.359 (Adjusted R^2^ = 0.290).

c. R^2^ = 0.462 (Adjusted R^2^ = 0.404).

### Field study

#### First experiment

**Figure 3:**
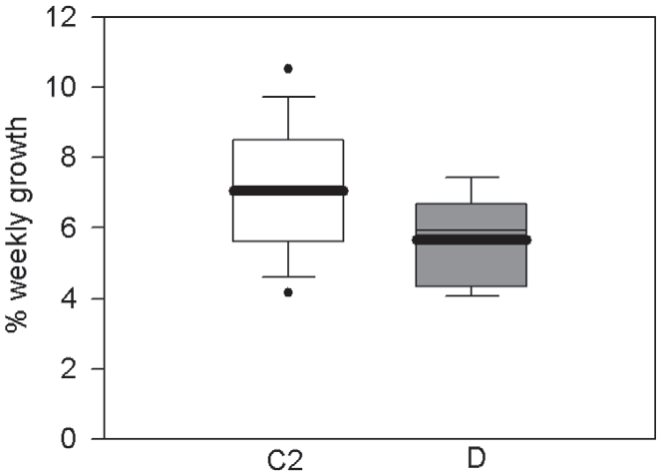
Growth of *Acropora millepora* with C2 and D symbionts in the field. Boxplots showing the significantly higher weekly growth rate of *Acropora millepora* colonies with type C2 symbionts compared to colonies with type D symbionts before a bleaching event. Boxes represent the weekly buoyant weight gain as a percentage of the initial buoyant weight of the colony. White boxes represent colonies with type C2 symbionts and grey boxes represent colonies with type D symbionts. Box boundaries represent the 75th and 25th percentiles. Thick lines within the boxplots represent the mean and thin lines represent the median. Whisker bars above and below the boxes represent the 95th and 5th percentiles. Missing and out of range values are not shown. Dots represent data that fall outside the confidence limits.

The first field growth experiment was conducted before a major bleaching event which affected the Keppel Islands in February 2006 [30]. At this time, the weekly growth rate of *A. millepora* colonies varied significantly with symbiont genotype (Figure 3, Table 3). The growth rate of D colonies was 38% lower than that of C2 colonies (p<0.05).

**Table 3:** ANOVA of weekly growth of *Acropora millepora* colonies before a bleaching event.

	SS	df	Mean Square	F	Sig.
Corrected Model	0.71^a^	1	0.71	4.9	0.032
Intercept	4.43	1	4.43	30.6	0.000
Type	0.71	1	0.71	4.9	0.032
Error	5.65	39	0.15		
Total	23.94	41			
Corrected Total	6.37	40			

a. R^2^ = 0.112 (Adjusted R^2^ = 0.089).

**Figure 4:**
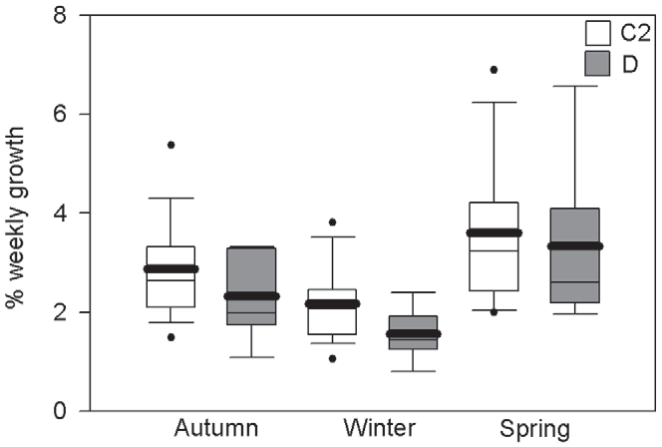
Seasonal growth of *Acropora millepora* colonies. The growth rate of colonies of *Acropora millepora* varied significantly with season in the year before a bleaching event. Boxes represent the weekly buoyant weight gain expressed as a percentage of the initial buoyant weight of the colony. White boxes represent colonies with type C2 symbionts and grey boxes represent colonies with type D symbionts. Box boundaries represent the 75th and 25th percentiles. Thick lines within the boxplots represent the mean and thin lines represent the median. Whisker bars above and below the boxes represent the 95th and 5th percentiles. Missing and out of range values are not shown. Dots represent data that fall outside the confidence limits.

The growth rates of *A. millepora* colonies in the field also varied significantly with season (Figure 4, Table 4). Growth rates were higher in spring and autumn than in winter. Growth rates were 27% higher in spring than in autumn (p<0.05) and 71% higher in spring than in winter (p<0.0.5) and 34% higher in autumn than in winter (p<0.05). There was no interaction between symbiont type and season.

**Table 4:** ANOVA of seasonal growth of *Acropora millepora* before a bleaching event.

	SS	df	Mean Square	F	Sig.
Season	3.21	2	1.61	34.6	0.000
Season * Type	0.11	2	0.06	1.2	0.311
Error(Season)	3.43	74	0.05		

#### Second experiment

**Figure 5:**
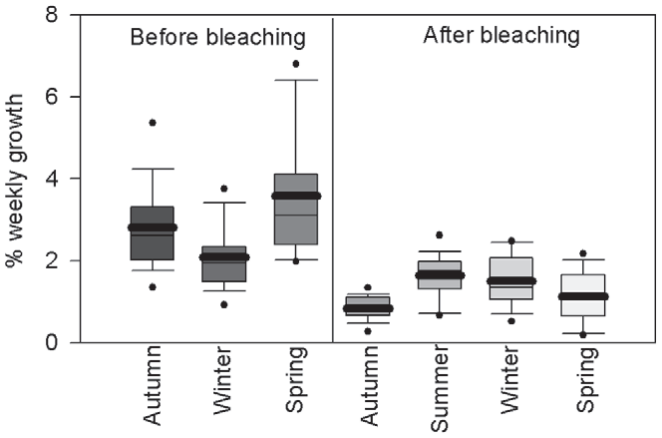
Growth of *Acropora millepora* colonies was severely affected by bleaching. The weekly growth rate of *Acropora millepora* colonies was severely affected by a bleaching event in January/February 2006. Boxes represent the weekly buoyant weight gain expressed as a percentage of the initial buoyant weight of the colony. Box boundaries represent the 75th and 25th percentiles. Thick lines within the boxplots represent the mean and thin lines represent the median. Whisker bars above and below the boxes represent the 95th and 5th percentiles. Missing and out of range values are not shown. Dots represent data that fall outside the confidence limits.

The bleaching event in early 2006 severely affected *A. millepora* growth rates irrespective of symbiont genotype (which correlated with bleaching severity). The growth of *A. millepora* colonies followed a similar seasonal pattern to the first study before the bleaching however colonies gained only half of the buoyant weight (p<0.05, Figure 5, Table 5). The highest growth rate in 2006 was in spring, six months after the bleaching event, 76% lower than the spring of 2005 before the bleaching (p<0.05, Table 5). Growth rate in autumn 2006 (12 months after the bleaching) was 46% lower than in autumn 2005 before bleaching (p<0.05). The winter 2006 growth rate (nearly 18 months after the bleaching), was 47% lower than the growth rate in the winter 2005 before the bleaching (p<0.05). The highest growth rates of *A. millepora* colonies after the bleaching event were in spring and the lowest growth rates were in autumn and winter.

**Table 5:** ANOVA of *Acropora millepora* growth during the combined field studies.

		SS	df	Mean Square	F	Sig.
Corrected Model	Hypothesis	52.57^a^	6	8.76	39.5	0.000
Intercept	Error	56.25	1	56.25	253.5	0.000
Season	Hypothesis	7.23	3	2.41	10.9	0.000
Bleaching	Error	41.96	1	41.96	189.1	0.000
Season * Bleaching	Hypothesis	5.84	2	2.92	13.2	0.000
Error	Error	47.48	214	0.22		
Total	Hypothesis	168.16	221			
Corrected Total	Error	100.05	220			

a. R^2^ = 0.525 (Adjusted R^2^ = 0.512).

Because of the low number of type C2 colonies left by the end of the first field experiment and the shifting background population of symbiont types, it was not possible to conduct a robust statistical analysis of the effects of symbiont type on growth. However, the overall growth rate of all the colonies in the second experiment (after bleaching) was 47% lower than the lowest growth rate (that of type D colonies) in the first experiment.

## Discussion

The growth rate of reef-building corals is likely to be significantly compromised by two separate and independent processes as they acclimatize to a warmer, more stressful, climate. Firstly, growth will be affected by symbiont population shuffling to more thermally tolerant types in response to thermal stress. The second process is the significant affect of bleaching stress itself on coral growth. Thermal stress involving bleaching is a key driver of symbiont shuffling. A community shift from thermally sensitive type C2 to thermally tolerant types D and C1 symbionts occurred in *A. millepora* colonies at Miall Island in the southern Great Barrier Reef following severe bleaching in 2006 [6]. Before the bleaching, colonies with predominantly type D symbionts had significantly lower growth rate than colonies with type C2 symbionts. Under normal (non-stressful) conditions, this shift is likely to have caused ∼38% lower growth in surviving colonies with predominantly type D symbionts. However, the effects of the bleaching event itself far out-weighed the effects of symbiont genotype, reducing growth in all colonies by 56% compared to the growth rate in the year before the bleaching. Even in corals with type D symbionts, that were apparently unaffected by heat stress at the peak of the bleaching event, growth was significantly reduced for at least 18 months after the bleaching event. It is unknown how long this stress event continued to impact coral growth as the experiment was terminated before there was any sign of recovery of growth rates to pre-bleaching levels.

This study provides conclusive evidence that symbiont genotype is a significant determinant of skeletal growth rate in *A. millepora* but suggests that although acclimation by symbiont shuffling may improve survival; it does not necessarily represent a long term benefit to host physiology, at least in terms of growth. Our finding that symbiont genotype influences adult coral growth supports previous studies that have demonstrated symbiont genotypic influences in juvenile corals in terms of their growth [14] and carbon acquisition [12]. While corals with thermally tolerant type D symbionts had a clear advantage over type C2 colonies in terms of survival [6], it is clear that at least in terms of skeletal growth, there are still significant heat stress effects on either the symbiont, the host or the holobiont. Our results suggest that acclimation by symbiont shuffling may not represent as beneficial an acclimatory response to warmer more stressful conditions as has previously been suggested. However, (and more importantly) if climate change causes reef-wide symbiont community change to more thermally tolerant types then there will be significant concurrent effects of symbiont change and thermal stress on reef growth and regeneration capacity.

Studies of scleractinian corals have previously found that bleaching can affect coral growth up to one year after bleaching. A correlation between growth variation and bleaching severity was found in *A. millepor*a colonies in a study by Baird and Marshall [31]. While moderately bleached colonies grew ∼20%, severely bleached colonies either remained the same size or shrank over a six week period following a natural bleaching event [31]. Two earlier studies of *Montastraea annularis* confirm the effects of bleaching on skeletal extension rates for up to a year after the event. The growth rate of bleached *M. annularis* colonies was reduced by ∼80% [32] and the growth rates of both bleached and unbleached colonies was reduced by 66–98% [33]. While it is difficult to compare measurements of buoyant weight gain to those of skeletal extension and colony area used in these studies, this confirms that severe bleaching can have a debilitating effect on coral growth rate up to a year and possibly longer following recovery. Following the 2006 bleaching at Miall Island, there was also evidence of a shift back to thermally sensitive C2 symbionts in the colonies within 18 months of the bleaching [6]. In this case, the effects of bleaching are expected to persist even longer than symbiont community change, suggesting that the growth differences that are a result of symbiont identity are transitory and relatively minor in comparison to the effects of heat damage to cells and photosystems. However, if climate change causes repeated anomalously warm summers, type D symbionts could become more widespread and permanent on reefs, resulting in the additive effects of these two processes acting to depress coral growth.

Some of the growth differences in *A. millepora* explants in the laboratory study can be explained by the lower symbiont densities of type D explants. However, since a correlation between *Symbiodinium* genotype and zooxanthellae densities has not previously been demonstrated [34], a significant proportion of the growth anomaly (∼13% at 23°C) can be attributed solely to symbiont genotype. This was confirmed by re-analyzing the laboratory growth data after standardizing the percentage growth rate of explants to zooxanthellae densities. The model results were consistent with the unstandardized growth data, confirming the link between symbiont identity and growth rate.

The growth differences of adult *A. millepora* with symbionts of contrasting thermal tolerance in the field and laboratory were not as dramatic as those found for juveniles of this species. Little *et al.* [14] found a far greater (200–300%) growth difference between juvenile *A. millepora* with type D and those with C1 symbionts and Mieog *et al.* [13] found a 50% growth difference. Three factors may be responsible for this disparity. The first two factors may be the effect of isometric scaling with the size of the subject [35] and the age of the coral tissues [36]. As tissues age, cell senescence causes a reduction in the proportion of the coral's energy allocated to growth and an increase in the energy allocated to reproduction. Older, larger colonies will partition less of their resources into growth than smaller, younger colonies. Coral recruits invest all their energy in tissue and skeletal growth in the absence of reproductive effort. The third rationale may lie in the identity of the symbionts compared in the studies. Little *et al.* [14] and Mieog *et al.* [13] compared *A. millepora* juveniles with type D to those with C1, whereas in the present study, adult corals with type D symbionts were compared to those with type C2 symbionts. The growth differences between adult *A. millepora* and juveniles of this species may assume more parity in the context of these factors. A further factor may lie in the effects of environmental variables which can influence growth rates in the field. Little *et al.* [14] studied growth at Magnetic Island whereas the present study took place at Miall Island, 800 km south of Magnetic Island (along-shelf distance). Mieog *et al.* [13] found differences at Magnetic Island but not in the Keppels; clearly demonstrating that environmental factors are at play. Irrespective of these factors, the range of growth values found between and within studies [31] indicate that further studies are required before it will be possible to fully quantify the effects of symbiont genotypes on growth in the field as corals acclimatize to climate change.

In terms of symbiont effects on growth, one possible explanation for the lower growth of type D compared to type C2 *A. millepora* under normal conditions may lie in the photokinetics of the symbionts. Rapid light curves (RLC's) were used to show a strong positive correlation between rETR_max_ of photosystem II and the incorporation of radio-labelled carbon into host tissues in juvenile *A. millepora* with type D and C1 symbionts [12]. Corals with type C1 symbionts had 87% higher rETR_max_ which correlated with more than double ^14^C incorporation rates. While a direct link between reduced photosynthetic carbon fixation of thermally tolerant symbionts and lower holobiont growth rate has yet to be demonstrated, one of the key mechanisms of thermal tolerance involves the composition and fluidity of the thylakoid membranes that house the photosystems [37]. Because these mechanisms in plants [38], [39] and micro algae [40] are linked to reduced growth, it is likely that the lower electron transport rate of type D symbionts in the study by Cantin *et al.* [12] may provide an explanation for the 38% lower growth in the field (under non-stressful conditions) in the present study. Lower photosynthetic function may help type D symbionts to cope with excess electrons and reactive oxygen species during heat stress, thereby maintaining normal photosynthetic function. However, the growth differences found in the field and laboratory in this study and those of Little *et al.* [14] suggest that heat tolerance comes at a cost to growth rates even at non-stressful temperatures.

In the second field study, *A. millepora* colonies with type D *Symbiodinium* had reduced growth in spite of retaining their symbionts during the bleaching event. There are a number of possible explanations for this. The photosynthetically fixed carbon from intact type D symbionts may not be available to their hosts for skeletal growth. This is the concept of type D symbionts as ‘greedy’ partners under stressful conditions. It is possible that surviving stress tolerant symbiont genotypes retain a greater portion of their photosynthetically fixed carbon for cell metabolism and repair, thereby ensuring their own survival but effectively starving the host coral. This would not occur under non-stressful conditions (i.e. before the bleaching) during which photokinetics remain a more likely explanation for the observed growth differences. A second explanation may be that during the warmer conditions of the summer bleaching event, in spite of retaining their symbionts, *A. millepora* with type D symbionts used more energy for respiration, which is positively correlated with temperature [41]. The increased respiratory demand would have occurred in both bleached and unbleached corals, resulting in reductions in growth in both C2 and D corals, irrespective of symbiont losses. This does not explain why growth rates remained low throughout the following year as temperatures became less stressful. The third explanation may be that type D symbionts had increased rates of photo-inhibition during the bleaching event which reduced carbon fixation. At high temperatures (e.g. 32°C), type D *Symbiodinium* has been shown to undergo protective photoinhibition [1]. Diversion of photon energy via photoprotective processes is a mechanism to cope with heat stress as temperature can damage the algal cell's capacity to repair proteins [42]. Photoinhibition mimics reduced habitat irradiance, reducing photosynthesis [1]. Finally, the effects of heat stress on ‘host factors’ may play a part in reducing photosynthate translocation to the corals which retained their type D symbionts [43]. It is likely that a combination of these mechanisms may cause the loss of skeletal growth in type D corals. What is clear is that in spite of increasing the heat tolerance of *A. millepora* colonies, hosting type D *Symbiodinium* does not protect the coral from the more subtle effects of the bleaching on processes such as growth.

The relative differences in growth rate of *A. millepora* in the field and the laboratory (nearly double) are likely to be caused by the interactions of influences such as light, morphology and changes in heterotrophic feeding behaviour. Theoretically at least, increased heterotrophy in the field [44], where zooplankton and particulate matter are available, should reduce incorporation of the heavier carbon isotope ^13^C into the coral skeleton because zooplankton and particulate matter are lower in δ^13^C (ratio ^13^C:^12^C relative to Vienna Peedee Belemnite Limestone Standard) than seawater [45]. In the laboratory, corals were supplied with filtered seawater which has comparatively low δ^13^C due to the absence of zooplankton. A second explanation is that the field growth rates incorporate both winter and summer rates. When compared to the growth rate of in spring (when temperatures were most similar to those in the laboratory), laboratory growth rates (at 23°C) assume greater parity.

This study has provided some insights into the synergistic effects and magnitude of symbiont genotype and thermal stress on coral growth. These two influences are likely to have implications to the future resilience and regeneration capacity of reefs. However more work is required to determine how applicable these effects are to other coral/algal associations and localities. The results of the field studies suggest that symbiont genotype will affect the growth rate of some reef corals, and that this will be compounded by the long-term effects of severe heat stress on these corals if they survive. Predictions of annual bleaching events within the next 30–50 years could result in more frequent disturbances which have the potential to shift the community composition of some reefs from hard-coral to macro-algae and soft coral-dominated communities [18]. Some of the most structurally important scleractinian corals may be able to acclimatize to gradually warmer waters by hosting thermally tolerant symbionts [5] but, the pressures of annual heat stress, ocean acidification and permanent symbiont changes on growth may act synergistically in compromising the competitiveness of these species to recover and compete between events.

## Materials and Methods

### Ethics statement

This study followed the guidelines of the Central Queensland University (CQU) Code of Conduct for Researchers and was conducted in accordance with the Great Barrier Reef Marine Park Authority and CQU Memorandum of Understanding and the Great Barrier Reef Marine Park Regulation (1983). The study did not require clearance by the CQU Animal Ethics Committee.

### Laboratory study

The growth rate of colonies with either C2 or D symbionts was measured in two studies. One study took place in the field on the reef slope at Miall Island in the Keppel region. To support the results of the field study, the second study took place under controlled laboratory conditions at two temperatures (23°C and 29°C). These temperatures represent the average stressful summer and non-stressful spring/autumn temperature ranges for corals at this site. The explants used in the laboratory experiment were sourced from the reef flat at Miall Island. The field experiment was repeated opportunistically following a natural bleaching event in February 2006 to further investigate the effect of bleaching on the growth differences between C2 and D corals.

#### Collection and maintenance of corals

In March 2005, 16 colonies of the Indo-Pacific stony coral *A. millepora*, Ehrenberg, 1834, with known *Symbiodinium* type C2 or type D were transplanted from the Keppel Islands region (a cool, clear southern inshore section of the Great Barrier Reef) to Magnetic Island (central Great Barrier Reef, ∼800 km north of Keppel). Corals were kept for a period of three months at Magnetic Island to allow recovery from transportation and acclimatization prior to the experiment. Temperatures ranged between 23°C and 27°C in the Keppels and between 24°C and 27°C at Magnetic Island during this time. Corals were kept on wire mesh racks at approximately the same depth that they were collected. In May 2005, the colonies were removed from the racks at Magnetic Island and transported to the Australian Institute of Marine Science (AIMS) where they were used for the growth experiment.

#### Experimental protocol

Six explants were cut from each of the 16 colonies (9 colonies with rDNA ITS1 type C2 and 7 colonies with ITS1 type D *Symbiodinium*) and distributed randomly and equally between three tanks (treatment replicates) within each of two temperature treatments (23°C and 29°C). Aerated seawater was supplied to the tanks at a flow rate of ∼1000 l h^−1^ and heated to the target temperatures (23°C and 29°C±1°C, mean ± S.D.). Coral explants were fixed to plastic stands with a cyanoacrylate-based adhesive (Loctite 454™ super glue gel) and then placed on elevated rotisseries. Each rotisserie was turned 180° twice daily to ensure even exposure to light and water flow. Corals were gradually acclimated for 10 days to light conditions in the tanks at the treatment temperatures. For the duration of the four week experiment, corals were supplied with 3.5 hours of shaded light (30–36 µmole photons m^−2^ s^−1^) followed by 5 hours of un-shaded light (87–107 µmole photons m^−2^ s^−1^), followed by another 3.5 hours of shaded light and 12 hours darkness each day to approximate their natural diurnal light cycle.

Light was provided by 10×400 W metal halide lamps (10,000°K colour temperature, BLV Germany) with a spectral quality suitable for coral photosynthesis. To monitor the health of explants with respect to the laboratory conditions, the dark-adapted maximum quantum yield of each explant was determined every second day by measuring Fv/Fm with a mini-PAM fluorometer (Heinz Walz, Germany) at the same time each morning after 8 hours of darkness. Measurements were made with a Diving-PAM fluorometer (Heinz Walz, Germany) sensor 5 cm underwater just above the coral explants with the tip of the fibre-optic probe touching the base of the explant surface on a vertical plane. Photosynthetically active radiation (PAR) measurements were made in the presence of a weak measuring light (F_0_) and then during a 1 s (8000 µmol photon m^−2^.s^−1^) saturating pulse of light (F_m_). Damping and gain were set at 2 and the measuring light was set at 1 s µmol photons m^−2^.s^−1^.

#### *Symbiodinium* identification

The predominant *Symbiodinium* type in the colonies used in both field and laboratory experiments was verified just before the start of the experiments using Single Stranded Conformational Polymorphism (SSCP) analysis of the Inter-transcribed Spacer Region 1 (ITS1) of algal nuclear ribosomal DNA as described in Jones *et al.* [30]. Only colonies with intense SSCP bands representing type C2 and type D (EU189443, EU1894505) were chosen for the studies although the presence of other types below 5% abundance is not ruled out [46].

#### Buoyant weight determination

Coral explants were weighed to three decimal places at the end of each week for four weeks to determine equivalent skeletal buoyant weight using the methods described in Jokiel *et al.* [47].

#### Zooxanthellae densities and pigments

To determine the influence of zooxanthellae densities and algal pigment concentrations on coral growth, explants were snap-frozen in liquid nitrogen and stored at −20°C immediately following the experiment. Frozen branches were stripped of tissue using an air gun and the resultant slurry was macerated with a tissue homogenizer for 20 s. The homogenate volume was recorded and a 9 ml aliquot was drawn off and preserved with 1 ml of formalin (32% w.w^−1^). Zooxanthellae counts were made on eight independent drops (0.0001 mm^3^) from each sample using a New Improved Nuebauer haemocytomer under a compound light microscope. Zooxanthellae numbers were standardized to coral tissue surface area using the 3D digital image analysis method described in Jones *et al.* [48].

A separate 10 ml aliquot was drawn from the remaining tissue homogenate and the algal pellet was separated from the host tissue by centrifugation (3000 g for 5 min) at 4°C. Chlorophyll was extracted overnight from the algal pellet using 100% methanol at 4°C. The first 10 samples were extracted three times to determine the extraction efficiency. Absorbance at 668 nm and 635 nm was measured with a spectrophotometer (Hitachi U-3200). Total branch chlorophyll *a* was calculated from the equation of Jeffrey and Haxo [49] after adjustment for extraction efficiency and standardized to algal cells.

### Field study

In March 2004, 43 pieces (15–20 cm) of *A. millepora* colonies from the Keppel region were cut from larger colonies from the reef flat and pruned to approximately similar sizes [36]. Colonies were genotyped in March 2004, at the start of the experiment using SSCP analysis of the algal nrDNA ITS1 region. Due to the low abundance of type D colonies at Miall Island when the study began, thirty six C2 colonies and only five D colonies were included in the first of the two field experiments. Initial buoyant weight measurements were made on the coral colonies in March 2004. Buoyant weight measurements (to the nearest gram) were performed by carefully transporting the colonies submerged in seawater to the weighing equipment a few 100 m from the study site. Colonies were carefully transported back to the study site after the buoyant weight measurements were completed and secured with plastic cable ties onto wire racks 75 cm above the sea bed at a depth of 3–4 m. Buoyant weight measurements were repeated seasonally every three months for a total of 9 months at the end of autumn (March to June 2005), winter (June to September 2005) and spring (September to December 2005).

The field growth experiment was repeated for another 12 months after a bleaching event in February 2006. Seven C2 and 15 D colonies were placed on the racks in May 2006 and allowed to recover from the bleaching until the experiment started in August 2006. The D colonies were sourced from the field while the C2 colonies were sourced from colonies in the first experiment that bleached but survived due to low abundance of C2 colonies in the field post-bleaching [6]. Symbiont genotypes were verified in May 2006 and then just before the start of the experiment in August 2006 using SSCP analysis. Only colonies with strong C2 or D SSCP bands were chosen for the experiment (verified by the intensity of the band) [46].

However, by the start of the study in August (3 months later), nearly all of the 22 colonies on the racks had undergone some change in symbiont proportions; gaining C2, D, or another thermally tolerant type, C1. The dynamic nature of the symbiont community after bleaching made a comparison of the growth rate of colonies as a function of symbiont genotypes difficult. Nevertheless, results from the second field experiment are included because they provide an insight into the overall growth performance of *A. millepora* pre-and post bleaching.

Colonies were weighed every three months at the end of spring (August to November 2006), summer (November to January 2007), autumn (January to May 2007) and winter (May to August 2007). Colonies used in the growth studies were not sampled for symbionts density or chlorophyll content to avoid compromising skeletal weight changes. Nearby C2 colonies showed ∼80% decline in symbiont densities following the bleaching. Nearby colonies also had lower algal chlorophyll *a* and *c_2_* content irrespective of symbiont genotype (data not shown).

### Statistical analysis

#### Laboratory study

To examine overall growth, the weekly buoyant weight measurements were expressed as a percentage of the initial buoyant weight of the explant and averaged over the four weeks of the study. Data for the percent average weekly buoyant weight gain of the explants were analyzed with a nested ANOVA using symbiont Type (fixed, two levels), Temperature (fixed, two levels), Tank (random, three levels, nested within temperature) as factors in the model. There were no significant differences between growth in the treatment tanks and data were averaged across the three tanks and the model re-run with an orthogonal ANOVA model using the fixed factors symbiont Type and Temperature. Unstandardized predicted values and standardized residuals were used to check the assumption of normality. Levene's test was used to verify homogeneity of variances.

To examine their influence on explant growth, data for zooxanthellae densities and chlorophyll *a* and *c_2_* concentrations in the laboratory experiment were analyzed with separate multivariate ANOVA's using symbiont Type (two levels) and Temperature (two levels) as fixed factors in the models and Tank (three levels) as a random factor nested within temperature. Unstandardized predicted values and standardized residuals were used to check the assumptions of normality. Levene's test was used to verify homogeneity of variances. Zooxanthellae densities and chlorophyll *a* and *c_2_* values were aggregated across all three treatment tanks. Zooxanthellae densities and algal cell chlorophyll *a* and *c_2_* concentrations were examined with an orthogonal multivariate ANOVA using Temperature (two levels) and Type (two levels) as fixed factors in the analysis.

#### Field study

To examine growth variation of *A. millepora* colonies with respect to symbiont type in the first field study, before the bleaching, a one-factor ANOVA was performed on the weekly growth rates using symbiont Type as the fixed, predictor variable (two levels). Growth for each colony was expressed as the weekly buoyant weight gain as a percentage of the initial buoyant weight of the colony at the start of the study. The assumption of normality was verified using plots of the unstandardized predicted values by the standardized residuals and Levene's test was used to verify the homogeneity of variances. Data were natural-log transformed to improve the normality of the distribution.

To examine the seasonal variations in growth of *A. millepora* colonies with different symbionts types in the first field experiment, data for the weekly buoyant weight gain during each three-month season were analyzed with a repeated-measures ANOVA using symbiont Type as the fixed variable (two levels) and Season (three levels) as the repeated measure in the model. The growth rate was expressed as the weekly buoyant weight gain of each colony in the study over the three month season as a percentage of the initial buoyant weight of the colony. The model residuals were examined to verify the validity of the assumption of normality and Levene's test was used to verify the homogeneity of variances. Data were natural-log transformed to improve the normality of the distribution.

To examine the variation in seasonal growth of *A. millepora* colonies as a result of the bleaching event in early 2006, the weekly growth rates for each season in the two studies were analyzed with ANOVA using the fixed factor Bleaching (before or after bleaching), and the random factor Season (three levels) as predictor variables. The growth rate was expressed as the weekly buoyant weight gain as a percentage of the initial buoyant weight of the colony. The assumption of normality was verified using plots of the unstandardized predicted values by the standardized residuals. Levene's test was used to verify the homogeneity of variances. Data were natural-log transformed to improve the normality of the distribution. Simple pair-wise comparisons were performed to further investigate significant differences in growth using Sidak's adjustment for multiple comparisons [50]. All statistical tests were completed using SPSS Version 15.0.
